# Performance Analysis of Constrained Loosely Coupled GPS/INS Integration Solutions

**DOI:** 10.3390/s121115983

**Published:** 2012-11-20

**Authors:** Gianluca Falco, Garry A. Einicke, John T. Malos, Fabio Dovis

**Affiliations:** 1 Istituto Superiore Mario Boella, Via P.C. Boggio 61, 10138 Torino, Italy; 2 CSIRO Exploration and Mining, Pullenvale, QLD 4069, Australia; E-Mails: garry.einicke@csiro.au (G.A.E.); john.malos@csiro.au (J.T.M.); 3 Politecnico di Torino, Department of Electronics and Telecommunications, Corso Duca degli Abruzzi, 24, 10129 Torino, Italy; E-Mail: fabio.dovis@polito.it

**Keywords:** loosely coupled integration, Kalman filter, constraints, GPS outages

## Abstract

The paper investigates approaches for loosely coupled GPS/INS integration. Error performance is calculated using a reference trajectory. A performance improvement can be obtained by exploiting additional map information (for example, a road boundary). A constrained solution has been developed and its performance compared with an unconstrained one. The case of GPS outages is also investigated showing how a Kalman filter that operates on the last received GPS position and velocity measurements provides a performance benefit. Results are obtained by means of simulation studies and real data.

## 1. Introduction

The error in an inertial system grows very quickly over time even if when an initial calibration procedure has been performed. Position error bias on startup also significantly affects position error over time. In fact, an initial calibration can correct short term errors only and the position error can become unacceptable after a very short period of time. In order to mitigate the error of inertial devices another sensor can be used in cooperation with the Inertial Measurement Unit (IMU). Typically, an absolute precise position estimate from a GPS receiver can be used to reset an Inertial Navigation System's (INS) solution or may be integrated with it by applying a data fusion algorithm (e.g., Kalman filter). The benefits of a GPS/INS integration are that the INS estimates can be corrected by the GPS data and that the INS can provide position and angle updates at a quicker rate than GPS. For highly dynamic vehicles such as missiles and aircraft, INS navigation solutions can interpolate between the GPS updates. Additionally, GPS signal losses may occur and the INS can continue to calculate position, velocity and orientation angles during outages. The two systems are complementary and are often employed together. Several approaches are possible for the integration of GPS and INS to provide a combined navigation solution. Such integration strategies differ on the type of information that is shared between the systems. There are four different categories of integration approaches: un-coupled [1], loosely coupled (LC) [2,3], tightly coupled (TC) [4,5], and ultra-tightly coupled (UTC) techniques [6–8]. The first method is the simplest integration of GPS and INS. The two systems operate independently, but when a GPS position and/or velocity measurement is available the IMU is reset. This method does not provide any performance enhancement. The second approach uses GPS position and velocity measurements in a Kalman filter that models INS error dynamics, while the third uses GPS estimates of Pseudoranges and Doppler and inertial estimates within a Kalman filter. In the UTC approach, outputs from the central navigation processor, after projection into satellite line-of-sight coordinates, are used to control the code and carrier replica signals for each satellite channel. On the other hand, a conventional tightly coupled GPS/INS system uses separate tracking loops for each satellite channel, which operate autonomously. As a result, the UTC design is considered more robust to jamming and vehicle dynamics.

In this work we address a loosely coupled approach. The paper investigates the performance of such an integration using both simulated and real measurements. For real tests we have used a Sirf-JP13 [9] and a Microstrain 3DM-Gx2 [10] modules as GPS receiver and IMU, respectively. In this work we have also considered the possibility to receive additional geographic information as an aiding to the position provided by the GPS receivers. This extra info can be delivered by a Google Map (GM) service if we have an embedded system equipped with GPS, IMU and a communication transceiver in order to establish an internet connection to download data from the GM service. In such a scenario we have developed a LC algorithm able to exploit additional position information when available. Furthermore, the case-study of an operational scenario in which GPS outages are experienced, has been analyzed. In such a case, a Kalman filter that leverages on the last received GPS measurement has been designed in order to reduce the error of INS-only navigation solution.

The paper is organized as follows: Section 2 describes the main characteristics of the INS mechanization equations and it provides the main features of the Kalman filter that will be applied in a loosely coupled integration. Results obtained through simulations and in a real scenario are presented. Section 3 deals with the improvements obtained by using known information to constrain the solution, such as the boundaries of the road along which the system is travelling, provided by an external aiding source such as Google Map. A description of the Kalman filter designed to include constraints information is also given. Performance comparisons are presented for an unconstrained loosely coupled system using simulated and real data.

Section 4 demonstrates the performance of a loosely coupled system in case of a 50-s-long GPS outage. An approach that uses a Kalman filter to reduce INS position error has been developed by exploiting the last available GPS position and velocity. Eventually, conclusions are drawn in Section 5.

## 2. Loosely Coupled GPS/INS Integration

### 2.1. Loosely Coupled and INS Equations

LC integration combines estimates from GPS and INS outputs, and such integration might be basically performed in two different ways. The first one, referred as open loop, estimates INS error by exploiting GPS information, and does not interfere with the operation of INS. The second approach, named closed loop, involves the use of a Kalman filter to mitigate INS errors.

**Figure 1.**
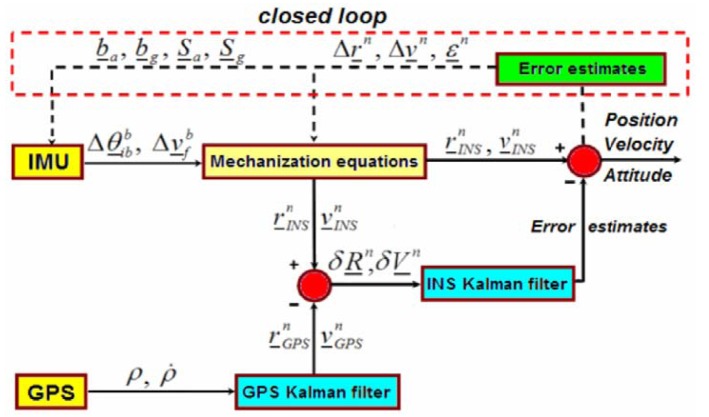
A closed-loop Loosely Coupled GPS/INS integration scheme [3].

In our work we have employed the second method where a Kalman filter calculates position and velocity error states to correct the INS solution. The block diagram of the closed-loop LC solution is shown in Figure 1.

Independent position and velocity estimates are calculated within a GPS receiver and are optionally filtered. Then, the output of this filter is used periodically as input to an INS filter. The second Kalman filter uses the difference between the GPS-derived positions, velocities and the ones computed by means of an INS device to get the error estimates.

The design of the Kalman filter for loosely coupled integration is described later in this Section. An INS filter generally consists of nine navigation error states, including three positions Δ*r̲^n^*, three velocities Δ*v̲^n^* and three attitude error states *ε̲^n^*, see [3,11–17]. For convenience, the symbols *x̲* and $\underline{\underline{x}}$ are used from this moment on to indicate a vector and matrix, respectively. Due to the presence of noise in the inertial sensor measurements, the system state vector needs to be enlarged depending on the inertial sensor's error characteristics.

The increased number of the error states could include bias error estimations both of the INS gyros and accelerometers (*b̲_g_* and *b̲_a_*) and/or the scale factor estimations (*S̲_g_* and *S̲_a_*). The output noise within accelerometer and gyro measurements may be represented as:

(1) $$\begin{array}{c} {\text{noise}}_{\text{acc}}=(1+{\underline{S}}_{a})\cdot \underline{f}+{\underline{b}}_{a}(t)+{\underline{w}}_{a}\\ {\text{noise}}_{\text{gyro}}=(1+{\underline{S}}_{g})\cdot \underline{f}+{\underline{b}}_{g}(t)+{\underline{w}}_{g} \end{array}$$

where:

- *f̲* is the correct acceleration or angular velocity (in the body frame);
- *S̲_a_*, *S̲_g_* are the scale factor of accelerometers and gyros;
- *b̲_a_*, *b̲_g_*, is the bias of accelerometers and gyros that can be considered constant over time *t*;
- *w̲_a_*, *w̲_g_* is the white noise component of accelerometers and gyros respectively.

**Figure 2.**
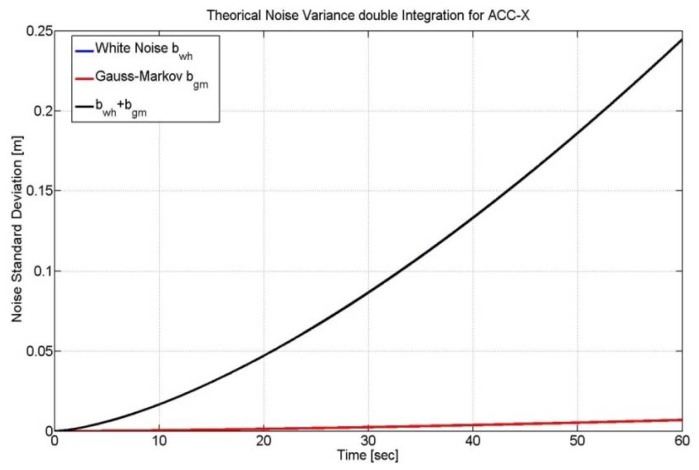
Theoretical Double Integration of different noise sources for a calibrated Gx2IMU.

In the paper we have modeled the accelerometers and gyroscopes' noises as white noise components (*i.e.*, *w̲_a_*, *w̲_g_*) and we did not consider the deterministic errors, such as the scale factor and the bias, since their contribution is negligible. Moreover, we have used both simulated and real data to model gyro and accelerometers errors. As far as the simulation test is concerned, we have developed a proper software in Matlab^®^ able to generate accelerometers and gyros raw measurements at different rates (e.g., sampling frequency at 500 or 100 Hz) and with different noise components. Furthermore, the simulator can provide information about the orientation angles (yaw, pitch and roll) of a vehicle that is moving along a trajectory. As far as the simulated path is concerned, we have implemented the same surveyed track of the real scenario in which the tests have performed, giving us the possibility to do accurate comparison between synthetic data and the real ones. In the case of real data we have utilized measurements from a 3DM-Gx2 MEMS-IMU. It has been argued in [11] that gyro and accelerometer errors of this device are dominated by white noise (see Figure 2). Therefore, a 9-state Kalman filter should be adequate for correcting inertial solutions.

**Figure 3.**
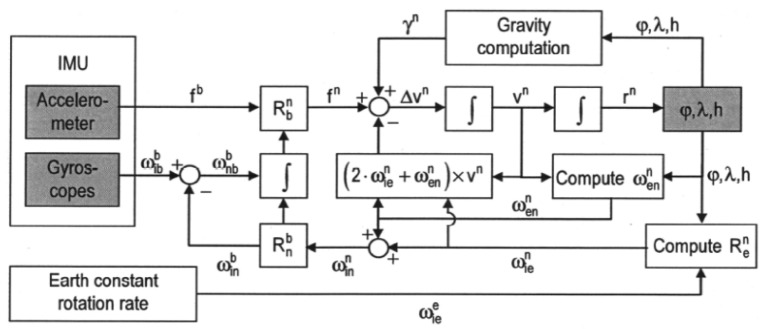
Scheme of the INS Mechanization equations [3].

The perturbation of the inertial navigation equations to obtain error states is detailed in [3]. A scheme that summarizes the overall n-frame INS processes is provided in Figure 3. A common orientation for Local Tangent Plane is the North-East-Down (NED) system defined as follows:

X^n^ horizontal axis in the direction of increasing latitude;
Y^n^ horizontal axis in the direction of increasing longitude;
Z^n^ to make a right-handed orthogonal coordinate system.

In the following we will refer to the NED coordinate system as n-frame.

The position in the n-frame is expressed in geodetic coordinates, namely

(2) $${\underline{r}}^{n}={\left[\varphi ,\lambda ,h\right]}^{T}$$

where φ, λ and *h* represent the latitude, longitude and altitude of the estimated user's position, expressed in radians and meters (for altitude) respectively.

The velocities in the n-frame are given by:

(3) $${\underline{v}}^{n}={\left[v_{N},v_{E},v_{D}\right]}^{T}$$

where *v_N_*, *v_E_*, *v_D_* are the velocities along North, East and Down coordinates and computed in m/s.

The motion of a vehicle can be described by equations that involve INS kinematics. The derivations of these equations can be broken up into three parts: position, velocity and attitude. A full derivation is reported in [12]. The position, velocity and attitude rates (from [3] and [13]) are given by:

(4) $$\left[\begin{array}{c} {\underline{\dot{r}}}^{n}\\ {\underline{\dot{v}}}^{n}\\ \underline{\dot{\Omega }} \end{array}\right]=\left[\begin{array}{c} {\underset{═}{D}}^{-1}\cdot {\underline{v}}^{n}\\ {\underset{═}{R}}_{b}^{n}\cdot {\underline{f}}^{b}-(2{\underline{\omega }}_{\text{ie}}^{n}+{\underline{\omega }}_{\text{en}}^{n})\times {\underline{v}}^{n}+{\underline{g}}^{n}\\ \underset{═}{R}\cdot \left({\underline{\omega }}_{\text{ib}}^{n}\right) \end{array}\right]$$

*D͇*^−1^ is a diagonal matrix defined as follows:

(5) $${\underset{═}{D}}^{-1}=\left[\begin{array}{ccc} \frac{1}{R_{M}+h} & 0 & 0\\ 0 & \frac{1\prime }{(R_{N}+h)\cos\varphi } & 0\\ 0 & 0 & -1 \end{array}\right]$$

where R_M_ is the radius of curvature in the meridian and R_N_ is the prime vertical at certain latitude expressed as:

(6) $$\begin{array}{c} R_{M}=\frac{a(1-e^{2})}{{\left(1-e^{2}\cdot \sin^{2}\varphi \right)}^{3/2}}\\ R_{N}=\frac{a}{{\left(1-e^{2}\cdot \sin^{2}\varphi \right)}^{1/2}} \end{array}$$

with a = 6378317.0 m and e = 0.0818 and where *f̲^b^* is the acceleration information in the body-frame and ${\underline{R}}_{b}^{n}$ is the frame rotation matrix from body to n-frame.

(7) $${\underline{R}}_{b}^{n}=\left[\begin{array}{ccc} \cos\psi \cos\theta & \cos\psi \sin\theta \sin\phi -\sin\psi \cos\varphi & \cos\psi \sin\theta \cos\phi +\sin\psi \sin\phi \\ \sin\psi \cos\theta & \sin\psi \sin\theta \sin\phi +\cos\psi \cos\phi & \sin\psi \sin\theta \cos\phi -\cos\psi \sin\phi \\ -\sin\theta & \cos\psi \sin\phi & \cos\theta \cos\phi \end{array}\right]$$

A more detailed explanation of the previous equations can be found in [3] and [17]. The vector Ω̲ = [*φ*, *θ*, *ψ*] consists of the Euler angles (Roll, Pitch and Yaw). The orientation angles are computed by exploiting the gyroscopes sensors. Other techniques are based on a blending of accelerometers and magnetometers to compute the attitude [18,19]. Although this last method is particularly suitable for low-cost MEMS IMUs whose gyroscopes are not sensitive to the Earth's rotation (for this reason the yaw can not be estimated properly through gyro-compassing techniques [20]), it requires, on the other hand, an additional Kalman filter to combine the measurements coming from the two sensors (*i.e.*, accelerometers and magnetometers). Therefore, we prefer to keep the integration level as simple as possible and we will design a unique GPS/INS Kalman filter where the yaw information is provided by the GPS receiver itself [21].

${\underline{\omega }}_{\text{ie}}^{n}$, ${\underline{\omega }}_{\text{en}}^{n}$ are the rotation vectors from the e-frame to the n-frame and the rate of change of latitude and longitude, respectively [3]:

(8) $$\begin{array}{c} {\underline{\omega }}_{\text{ie}}^{n}=[\begin{array}{ccc} \omega_{e}\cos\varphi & 0 & -\omega_{e}\sin\varphi \end{array}]\\ {\underline{\omega }}_{\text{ie}}^{n}=\left[\begin{array}{c} \frac{v_{E}}{R_{N}+h}\\ -\frac{v_{N}}{R_{M}+h}\\ \frac{-v_{E}\tan\varphi }{R_{N}+h} \end{array}\right] \end{array}$$

where (*ω_e_* ≈ 7.2921155·10^5^ rad/s) is the magnitude of the Earth rotation rate.

*g̲^n^* is the local gravity vector and *R͇* is the transformation matrix from the body-axes angular rates to the Euler angle angular rates and is given by [3] as:

(9) $$\underset{=}{R}=\left[\begin{array}{ccc} 1 & \sin\phi \tan\theta & \cos\phi \tan\theta \\ 0 & \cos\phi & -\sin\phi \\ 0 & \cos\theta \sin\phi & \cos\theta \cos\phi \end{array}\right]$$

${\underline{\omega }}_{ib}^{b}$ represents the raw measurement vector of the gyros sensors in the bodyframe [3].

Eventually, the equations describing the error dynamics are obtained by perturbing the kinematic Equation (4). These error equations are required in the construction of the INS/GPS Kalman filter. The perturbation of the position, velocity and Euler angles can be written as:

(10) $$\begin{array}{c} {\underline{\hat{r}}}^{n}={\underline{r}}^{n}+{\underline{\delta r}}^{n}.\\ {\underline{\hat{v}}}^{n}={\underline{v}}^{n}+{\underline{\delta v}}^{n}.\\ {\underline{\hat{\Omega }}}^{n}={\underline{\Omega }}^{n}+{\underline{\delta \Omega }}^{n}. \end{array}$$

The linearized position error is given by:

(11) $$\begin{array}{c} {\underline{\delta \dot{r}}}^{n}={\underset{═}{F}}_{\text{rr}}\delta {\underline{r}}^{n}+{\underset{═}{F}}_{\text{rv}}\delta {\underline{v}}^{n}.\\ {\underset{═}{F}}_{\text{rr}}=\left[\begin{array}{ccc} 0 & 0 & \frac{-v_{N}}{{\left(R_{M}+h\right)}^{2}}\\ \frac{v_{E}\sin\phi }{(R_{N}+h)\cos^{2}\phi } & 0 & \frac{-v_{E}}{{\left(R_{N}+h\right)}^{2}\cos\phi }\\ 0 & 0 & 0 \end{array}\right]\\ {\underset{═}{F}}_{\text{rv}}={\underset{═}{D}}^{-1} \end{array}$$

The velocity error is given by:

(12) $$\begin{array}{c} {\underline{\delta \dot{v}}}^{n}={\underset{═}{F}}_{\text{vr}}\delta {\underline{r}}^{n}+{\underset{═}{F}}_{\text{vv}}\delta {\underline{v}}^{n}+\left({\underline{f}}^{n}\times \right)\delta {\underline{\Omega }}^{n}+{\underset{═}{R}}_{b}^{n}\delta {\underline{f}}^{b}\\ {\underset{═}{F}}_{\text{vr}}=\left[\begin{array}{ccc} -2v_{E}\omega_{e}\cos\varphi -\frac{{v_{E}}^{2}}{(R_{N}+h)\cos^{2}\varphi } & 0 & \frac{-v_{N}v_{D}}{{\left(R_{M}+h\right)}^{2}}+\frac{v_{E}^{2}\tan\varphi }{{\left(R_{N}+h\right)}^{2}}\\ 2\omega_{e}(v_{N}\cos\varphi -v_{D}\sin\varphi )+\frac{v_{E}v_{N}}{(R_{N}+h)\cos^{2}\varphi } & 0 & \frac{-v_{E}(v_{D}+v_{N}\tan\varphi )}{{\left(R_{N}+h\right)}^{2}}\\ 2v_{E}\omega_{e}\sin\varphi & 0 & \frac{v_{E}^{2}}{{\left(R_{N}+h\right)}^{2}}+\frac{v_{N}^{2}}{{\left(R_{M}+h\right)}^{2}}-\frac{2\gamma }{R+h} \end{array}\right]\\ \gamma =\gamma_{0}{\left(\frac{R}{R+h}\right)}^{2}\text{withR}=\sqrt{R_{M}R_{N}}\\ {\underset{═}{F}}_{\text{vv}}=\left[\begin{array}{ccc} \frac{v_{D}}{R_{M}+h} & 2\omega_{e}\sin\varphi -2\frac{v_{E}\tan\varphi }{R_{N}+h} & \frac{v_{N}}{R_{M}+h}\\ 2\omega_{e}\sin\varphi +\frac{v_{E}\tan\varphi }{R_{N}+h} & \frac{v_{D}+v_{N}\tan\varphi }{R_{N}+h} & 2\omega_{e}\cos\varphi +\frac{v_{E}}{R_{N}+h}\\ -2\frac{v_{N}}{R_{M}+h} & -2\omega_{e}\cos\varphi -2\frac{v_{E}}{R_{N}+h} & 0 \end{array}\right] \end{array}$$

The attitude error can be written as:

(13) $$\begin{array}{c} {\underline{\delta \dot{\Omega }}}^{n}=={\underset{═}{F}}_{\text{er}}\delta {\underline{r}}^{n}+{\underset{═}{F}}_{\text{ev}}\delta {\underline{v}}^{n}-\left(\omega_{\text{in}}^{n}\times \right){\underline{\Omega }}^{n}-{\underset{═}{R}}_{b}^{n}\delta {\underline{\omega }}_{ib}^{b}.\\ {\underset{═}{F}}_{\text{er}}=\left[\begin{array}{ccc} -\omega_{e}\sin\varphi & 0 & \frac{-v_{E}}{{\left(R_{N}+h\right)}^{2}}\\ 0 & 0 & \frac{-v_{N}}{{\left(R_{M}+h\right)}^{2}}\\ -\omega_{e}\cos\varphi -\frac{-v_{E}}{(R_{N}+h)\cos^{2}\varphi } & 0 & \frac{v_{E}\tan\varphi }{{\left(R_{N}+h\right)}^{2}} \end{array}\right]\\ {\underset{═}{F}}_{\text{ev}}=\left[\begin{array}{ccc} 0 & \frac{1}{R_{N}+h} & 0\\ \frac{-1}{R_{M}+h} & 0 & 0\\ 0 & \frac{\tan\varphi }{R_{N}+h} & 0 \end{array}\right] \end{array}$$

Details about Equations (10)–(13) can be found in [3,11–17].

### 2.2. Loosely Coupled Kalman Filter

In this subsection we recall the traditional design of an error state Kalman filter for a loosely coupled GPS/INS application. An n-frame error state model [3] is given by:

(14) $$\left[\begin{array}{c} \delta {\underline{\dot{r}}}^{n}\\ \delta {\underline{\dot{v}}}^{n}\\ \delta {\underline{\dot{\Omega }}}^{n} \end{array}\right]=\left[\underset{F}{\underset{︸}{\begin{array}{ccc} {\underset{═}{F}}_{\text{rr}} & {\underset{═}{F}}_{\text{rv}} & 0_{3\times 3}\\ {\underset{═}{F}}_{\text{vr}} & {\underset{═}{F}}_{\text{vv}} & \left({\underline{f}}^{n}\times \right)\\ {\underset{═}{F}}_{\text{er}} & {\underset{═}{F}}_{\text{ev}} & -({\underline{\omega }}_{\text{in}}^{n}\times ) \end{array}}}\right]\cdot \left[\begin{array}{c} \delta {\underline{r}}^{n}\\ \delta {\underline{v}}^{n}\\ \delta {\underline{\Omega }}^{n} \end{array}\right]+\underline{w}.$$

where all the sub-matrices *F͇_xx_* are the ones as stated in Equations (10)–(13). *f̲^n^* is the raw measurements of accelerometers expressed in n-frame whereas ${\underline{\omega }}_{\text{in}}^{n}$ is the raw information of the gyros sensors in the n-frame too. Eventually, parameters *w̲* indicate the noise components of gyros and accelerometers, respectively. As previously explained in Section 2.1, only white noise has been considered. The discrete-time analogue of (14) is expressed as:

(15) $${\underline{\Phi }}_{K}=e^{\underline{F}(t_{k})\Delta t}=\underset{═}{I}+\underset{═}{F}(t_{k})\Delta t+\frac{{\left(\underset{═}{F}(t_{k})\Delta t\right)}^{2}}{2}+h.o.t$$

where *h.o.t.* means higher order terms that can be neglected for the computation.

The covariance matrix *Q_K_* associated to the discrete-time noise vector *w̲* can be determined by the approximate expression [14]:

(16) $${\underset{═}{Q}}_{K}\approx \frac{1}{2}\left[{\underline{\Phi }}_{K}\underset{═}{G}(t_{K})\underset{═}{Q}(t_{K}){\underset{═}{G}}^{T}(t_{K})+\underset{═}{G}(t_{K})\underset{═}{Q}(t_{K}){\underset{═}{G}}^{T}(t_{K}){\underline{\Phi }}_{K}^{T}\right]\cdot \Delta t$$

where Δt is the sampling time that we set equal to 1 s and *G͇* is a matrix equal to:

(17) $$\underset{═}{G}=\left[\begin{array}{cc} 0_{3\times 3} & 0_{3\times 3}\\ R_{b}^{n} & 0_{3\times 3}\\ 0_{3\times 3} & -R_{b}^{n} \end{array}\right].$$

*Q͇* is a diagonal matrix representing the white noise on the accelerometers *q̲_a_* and *q̲_g_* gyros that can be stated as:

(18) $$\underset{═}{Q}=[\begin{array}{cc} \text{diag}({\underline{q}}_{a}) & 0_{3\times 3}\\ 0_{3\times 3} & \text{diag}({\underline{q}}_{g}) \end{array}]\cdot$$

In a loosely-coupled integration approach, the filter measurement is the difference between the INS and the GPS navigation solutions. The measurement vector is given by:

(19) $$\underline{z}=\left[\begin{array}{c} \delta {\underline{R}}^{n}\\ \delta {\underline{V}}^{n} \end{array}\right]=\left[\begin{array}{cc} {\underline{r}}_{\text{INS}}^{n} & -{\underline{r}}_{\text{GPS}}^{n}\\ {\underline{v}}_{\text{INS}}^{n} & -v_{\text{GPS}}^{n} \end{array}\right]=\left[\begin{array}{c} \varphi_{\text{INS}}-\varphi_{\text{GPS}}\\ \lambda_{\text{INS}}-\lambda_{\text{GPS}}\\ h_{\text{INS}}-h_{\text{GPS}}\\ {\underline{v}}_{\text{INS}}^{n}-{\underline{v}}_{\text{GPS}}^{n} \end{array}\right].$$

Following the approach suggested in [3], since φ and λ are in radians and their values are very small, we multiply the first two rows of Equation (19) by (*R_M_* + *h*) and (*R_N_* + *h*)cos *φ* to obtain:

(20) $$\underline{z}=\left[\begin{array}{c} \left(R_{M}+h)(\varphi_{\text{INS}}-\varphi_{\text{GPS}}\right)\\ \left(R_{N}+h)\cos\varphi (\lambda_{\text{INS}}-\lambda_{\text{GPS}}\right)\\ h_{\text{INS}}-h_{\text{GPS}}\\ {\underline{v}}_{\text{INS}}^{n}-{\underline{v}}_{\text{GPS}}^{n} \end{array}\right].$$

The design matrix becomes:

(21) $$\underset{═}{H}=\left[\begin{array}{ccccccccc} R_{M}+h & 0 & 0 & 0 & 0 & 0 & 0 & 0 & 0\\ 0 & (R_{N}+h)\cos\varphi & 0 & 0 & 0 & 0 & 0 & 0 & 0\\ 0 & 0 & 1 & 0 & 0 & 0 & 0 & 0 & 0\\ 0 & 0 & 0 & 1 & 0 & 0 & 0 & 0 & 0\\ 0 & 0 & 0 & 0 & 1 & 0 & 0 & 0 & 0\\ 0 & 0 & 0 & 0 & 0 & 1 & 0 & 0 & 0 \end{array}\right].$$

Finally, the measurement noise covariance matrix is calculated as:

(22) $$\underset{═}{R}=\left[\begin{array}{cccccc} {\left(R_{M}+h\right)}^{2}\cdot \sigma_{\varphi }^{2} & 0 & 0 & 0 & 0 & 0\\ 0 & {\left(R_{N}+h\right)}^{2}\cdot \cos^{2}\varphi \cdot \sigma_{\lambda }^{2} & 0 & 0 & 0 & 0\\ 0 & 0 & \sigma_{h}^{2} & 0 & 0 & 0\\ 0 & 0 & 0 & \sigma_{V_{N}}^{2} & 0 & 0\\ 0 & 0 & 0 & 0 & \sigma_{V_{E}}^{2} & 0\\ 0 & 0 & 0 & 0 & 0 & \sigma_{V_{D}}^{2} \end{array}\right].$$

The value of each element in the diagonal matrix R depends on the accuracy of the GPS estimates. A detailed description of Kalman filtering combining models with measurements is reported in [15]. However, since we use a feedback loosely coupled approach, the error state vector is set to zero after every measurement updates [16,17,22].

### 2.3. Results

**Figure 4.**
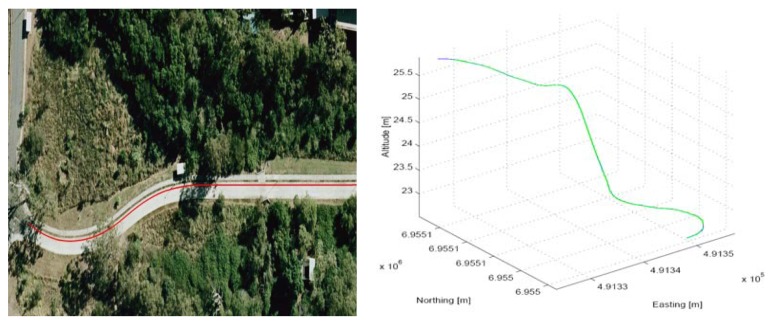
Test track view and 3D plot.

Tests were carried out along a surveyed track located within CSIRO's site in Pullenvale, QLD, Australia. A 3-D plot of the test track and is shown in Figure 4.

We investigated the algorithm's performance using both simulated data and real data. In the first case we have generated synthetic gyros and accelerometers values along a path that perfectly matches the realistic scenario. This simulation will be used as a term of comparison with the results we obtained by working with real GPS and INS measurements on the field.

We have also corrupted the simulated data, representing the inertial accelerometer and gyro, with noise sources resembling the typical characteristics of a low-cost MEMS IMU, as in Table 1.

Simulated GPS positions and velocities' estimates were also generated, and the variances of those measurements were set equal to the values reported in Table 2.

**Figure 5.**
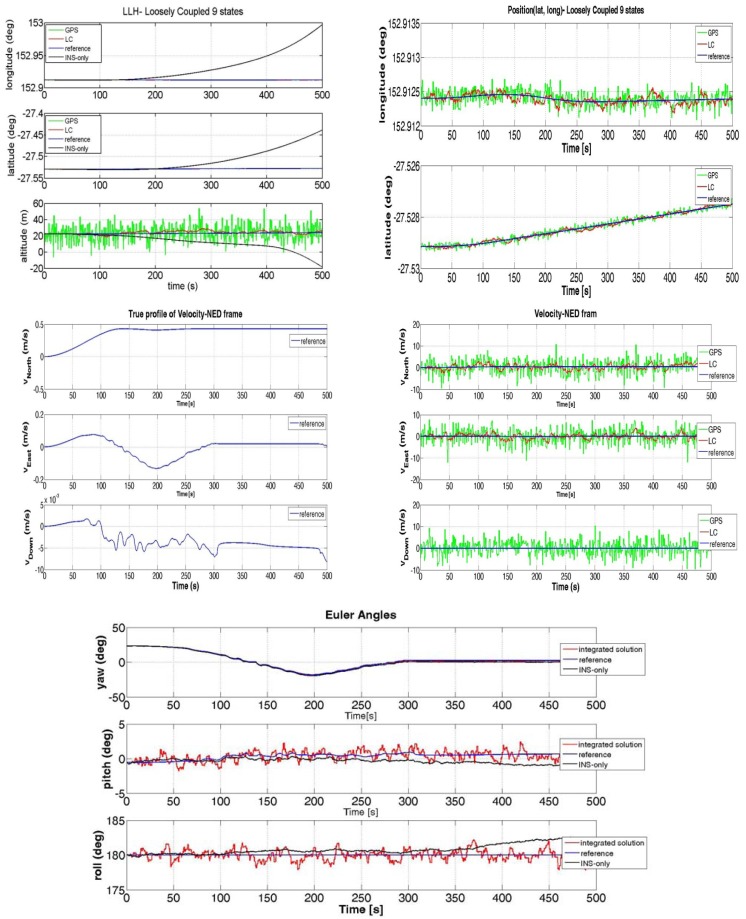
LC performance obtained by Simulation: Position (LLH), Velocity and Euler Angles.

The variances of Table 2 refer to the case of unfiltered GPS measurements. By utilizing the synthetic GPS and INS data we were able to run the LC algorithm and the results can be seen in Figure 5.

We have repeated the test, by using this time, data directly read from the GPS and the IMU sensors:

- raw gyro and accelerometer outputs from a 3DM-Gx2 INS after an initial calibration,
- positions and velocities' estimates from a Sirf JP13- Falcom GPS receiver.

In our LC GPS/INS implementation, the process noise covariance, Q, was estimated through the Allan Variance technique which is detailed in [11]. The components of the measurement noise covariance, R, were selected as summarized in Table 3.

**Figure 6.**
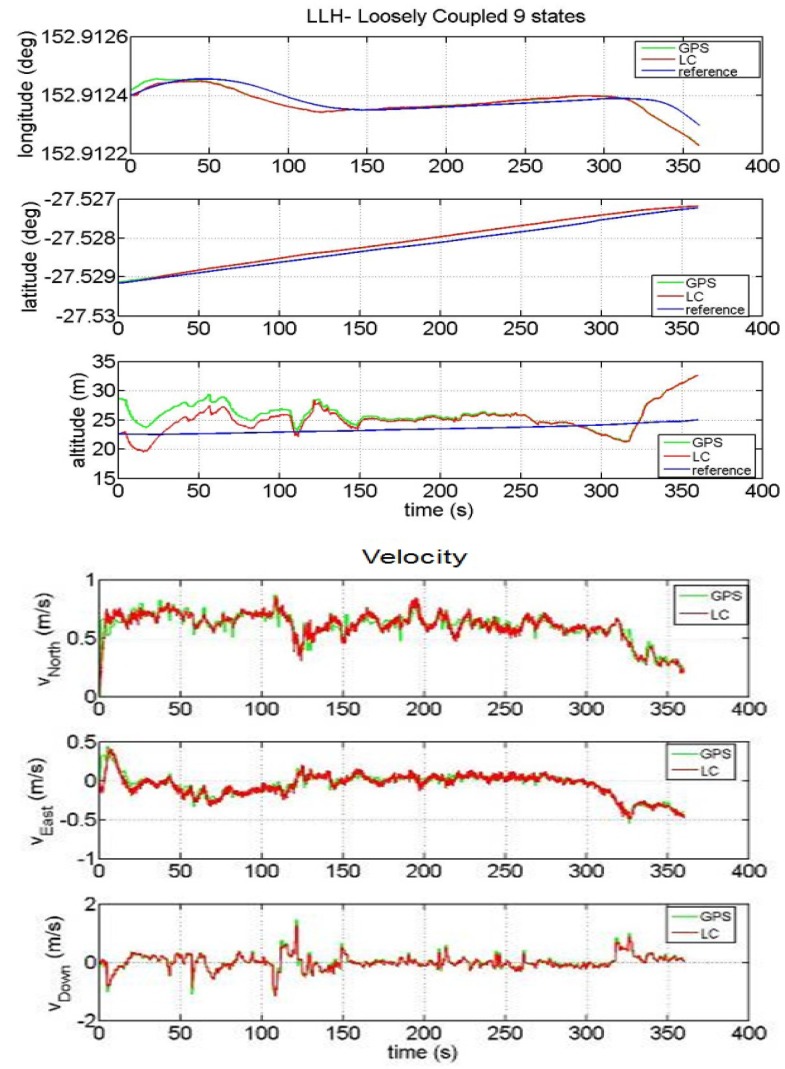

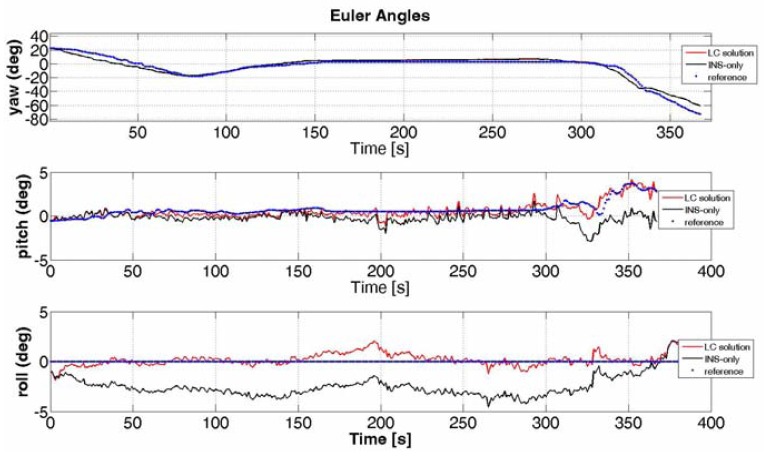
LC performance obtained with real data: Position (LLH), Velocity and Euler Angles.

The results of such a GPS/INS hybridization technique are plotted in Figure 6.

It is clear from Figure 5 that the performance of the LC integration solution (red line) significantly improves the performance of the INS-only solution (black line). In fact, the LC approach trusts the GPS estimates (when they are available) and does not allow the INS navigation solution to drift. The main difference between the synthesized measurements and the real ones is that the positions obtained by the simulated GPS are centred on the reference track and the deviations are modelled as white noise. On the other hand, in a real scenario, the user's position estimations at consecutive time instants are correlated because they are processed within the GPS receiver by a proper Kalman filter. Such measurements can have a bias offset with respect to the surveyed path (as it can be noted in Figure 6). This fact can be explained by considering that a GPS system has an accuracy that depends on several factors: quality of the GPS receiver, the algorithm used to compute the Position-Velocity-Time (PVT) with or without carrier phase information, the effect of ionosphere compensation in the PVT estimation, the number of visible satellites when the test is performed. Another aspect that is clear by observing Figures 5 and 6 is the different time required to the vehicle to complete the path when simulated or real data have been used. This fact should not be surprising if we consider the velocity profile of the two figures. By comparing the velocity along the eastern and northern directions, we can notice how the estimated speed is higher when real GPS data are applied with respect to the case of simulated data. As a consequence, the time necessary to run the trajectory will be shorter. Although the solution provided by GPS is sufficiently accurate (e.g. notice in Figure 6 the improvement in the Euler angles' estimation when the INS is aided by the GPS), it is still unable to fulfil the requirements of continuity and reliability in many situations. In order to reduce the error of the GPS we have designed a LC integration scheme that uses a Kalman filter with some constraints. This aspect will be described in details in the next Section and details on constrained Kalman filters can be found in [23–26].

## 3. Loosely Coupled Integration Using Constraints

### 3.1. Performance Assessment

**Figure 7.**
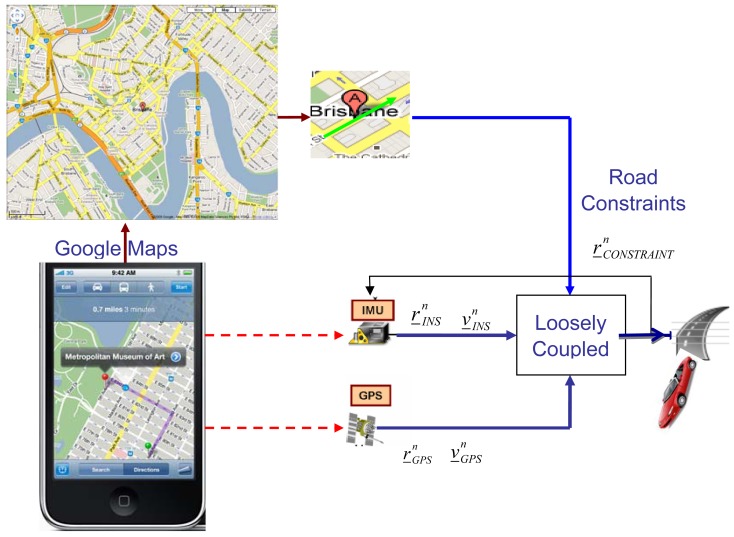
A closed-loop Loosely Coupled GPS/INS integration scheme with constraint.

It is well known that GPS estimation is affected by a certain error that is strongly dependent on the number of satellites available for the PVT estimation. Thus, as a consequence, the LC solution trusts the GPS position and velocity estimates in a way proportional to number of satellites in view. Therefore, the overall performance of the LC solution will leverage on the availability of the GPS updates. In order to improve the performance with respect to the results shown in Figures 5 and 6 we have designed a Kalman filter that exploits additional aiding information. In particular, we have considered an external source that provides some additional geographical data. For instance, such pieces of information can be obtained by the Google Maps service, as shown in the block diagram depicted in Figure 7. Such an implementation requires that the user is equipped with a smartphone designed to embed a GPS receiver, accelerometers and gyros sensors as well as a communication transceiver able to establish a wireless internet connection. In this way the user is able to receive information about the road being travelled, as provided by the Google Maps (GM) service.

As far as the accuracy of GM is concerned, the last improvements in terms of position precision developed by Google on GE and GM can be found in [27]. It has been shown in [28] that comparing GM and Google Earth (GE), the difference between the two mapping systems is of 2.5 m. On the other hand, when he repeated the test by plotting the point on high-accuracy map he noticed a difference of 10 m with respect to the GM.

This means the GM can not be used for systems that require a high level of accuracy but it could be fine for mass-market applications. Another concern of using a Google Maps service is the process of receiving the map information should be quick enough to be applied in real-time and the internet connection should always be available for all the duration of the test. If this happens we can build up a more complex LC scheme that also integrates street constraints to estimate the user's position and velocity.

**Figure 8.**
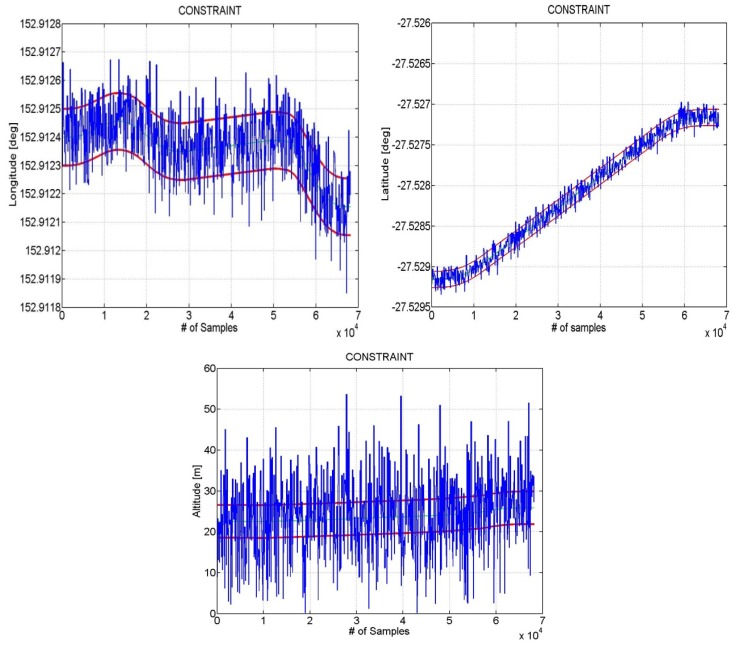
Example of constraints on position. The boundaries of the street are shown (red lines) and the user position has been approximated as affected by a white noise (blue).

In Figure 8 an example of results obtained using as constraints the boundaries of the road is shown. The blue line represents a simulated GPS position estimation over time whereas the red line indicates the boundary of the path the user is driving along. Both the GPS and constraint information are expressed in latitude, longitude and altitude coordinate respectively. In this example we have supposed the street is 10meters wide in both latitude and longitude. As for the altitude we have considered that the GPS estimation can be acceptable only when it falls within an interval of 4 meters with respect to the altitude information received by Google Maps. The boundaries of the hypothetical road have been computed setting the offsets Δ*n* = 10 [*m*], Δ*e* = [*m*] and then obtaining the Coordinates offsets (in radians) $\Delta \varphi =\frac{\Delta n}{R}$ and $\Delta \lambda =\frac{\Delta e}{R\cos\left(\frac{\pi \varphi }{180}\right)}$, where R = 6378137 [m] is the Earth radius and where *φ*, *λ* are the latitude and longitude coordinates as stated in Equation (2).

The offset in the position in decimal degrees is obtained as $\varphi_{\text{off}}=\varphi +\Delta \varphi \frac{180}{\pi }$ and $\lambda_{\text{off}}=\lambda +\Delta \lambda \frac{180}{\pi }$.

Constraint information can be added by increasing the dimensions of the output mapping matrix *H͇* of Equation (21) and the measurements *z̲* of Equation (20) as:

(23) $$\underline{z}=\left[\begin{array}{c} \left(R_{M}+h)(\varphi_{\text{INS}}-\varphi_{\text{GPS}}\right)\\ \left(R_{N}+h)\cos\varphi (\lambda_{\text{INS}}-\lambda_{\text{GPS}}\right)\\ h_{\text{INS}}-h_{\text{GPS}}\\ {\underline{v}}_{\text{INS}}^{n}-{\underline{v}}_{\text{GPS}}^{n}\\ \left(R_{M}+h)(\varphi_{\text{INS}}-\varphi_{\text{CONSTRAINT}}\right)\\ \left(R_{N}+h)\cos\varphi (\lambda_{\text{INS}}-\lambda_{\text{CONSTRAINT}}\right)\\ h_{\text{INS}}-h_{\text{CONSTRAINT}} \end{array}\right].$$

(24) $$\underset{═}{H}=\left[\begin{array}{ccccccccc} R_{M}+h & 0 & 0 & 0 & 0 & 0 & 0 & 0 & 0\\ 0 & (R_{N}+h)\cos\varphi & 0 & 0 & 0 & 0 & 0 & 0 & 0\\ 0 & 0 & 1 & 0 & 0 & 0 & 0 & 0 & 0\\ 0 & 0 & 0 & 1 & 0 & 0 & 0 & 0 & 0\\ 0 & 0 & 0 & 0 & 1 & 0 & 0 & 0 & 0\\ 0 & 0 & 0 & 0 & 0 & 1 & 0 & 0 & 0\\ R_{M}+h & 0 & 0 & 0 & 0 & 0 & 0 & 0 & 0\\ 0 & (R_{N}+h)\cos\varphi & 0 & 0 & 0 & 0 & 0 & 0 & 0\\ 0 & 0 & 1 & 0 & 0 & 0 & 0 & 0 & 0 \end{array}\right]$$

(25) $$R=\left[\begin{array}{ccccccccc} {\left(R_{M}+h\right)}^{2}\cdot \sigma_{\phi |\text{GPS}}^{2} & 0 & 0 & 0 & 0 & 0 & 0 & 0 & 0\\ 0 & {\left(R_{N}+h\right)}^{2}\cdot \cos^{2}\phi \cdot \sigma_{\lambda |\text{GPS}}^{2} & 0 & 0 & 0 & 0 & 0 & 0 & 0\\ 0 & 0 & \sigma_{h|\text{GPS}}^{2} & 0 & 0 & 0 & 0 & 0 & 0\\ 0 & 0 & 0 & \sigma_{V_{N|\text{GPS}}}^{2} & 0 & 0 & 0 & 0 & 0\\ 0 & 0 & 0 & 0 & 0 & \sigma_{V|{\text{GPS}}_{E}}^{2} & 0 & 0 & 0\\ 0 & 0 & 0 & 0 & 0 & 0 & \sigma_{V|{\text{GPS}}_{E}}^{2} & 0 & 0\\ 0 & 0 & 0 & 0 & 0 & 0 & {\left(R_{M}+h\right)}^{2}\cdot \sigma_{\phi |\text{CONS}}^{2} & 0 & 0\\ 0 & 0 & 0 & 0 & 0 & 0 & 0 & {\left(R_{N}+h\right)}^{2}\cdot \cos^{2}\phi \cdot \sigma_{\lambda |\text{CONS}}^{2} & 0\\ 0 & 0 & 0 & 0 & 0 & 0 & 0 & 0 & \sigma_{h|\text{CONS}}^{2} \end{array}\right]$$

As previously explained also in this case constraint information are computed in LLH coordinate system. The variances of the map measurements need to be well-selected so that the resulting position estimates fall within the constraint boundaries.

### 3.2. Results

**Figure 9.**
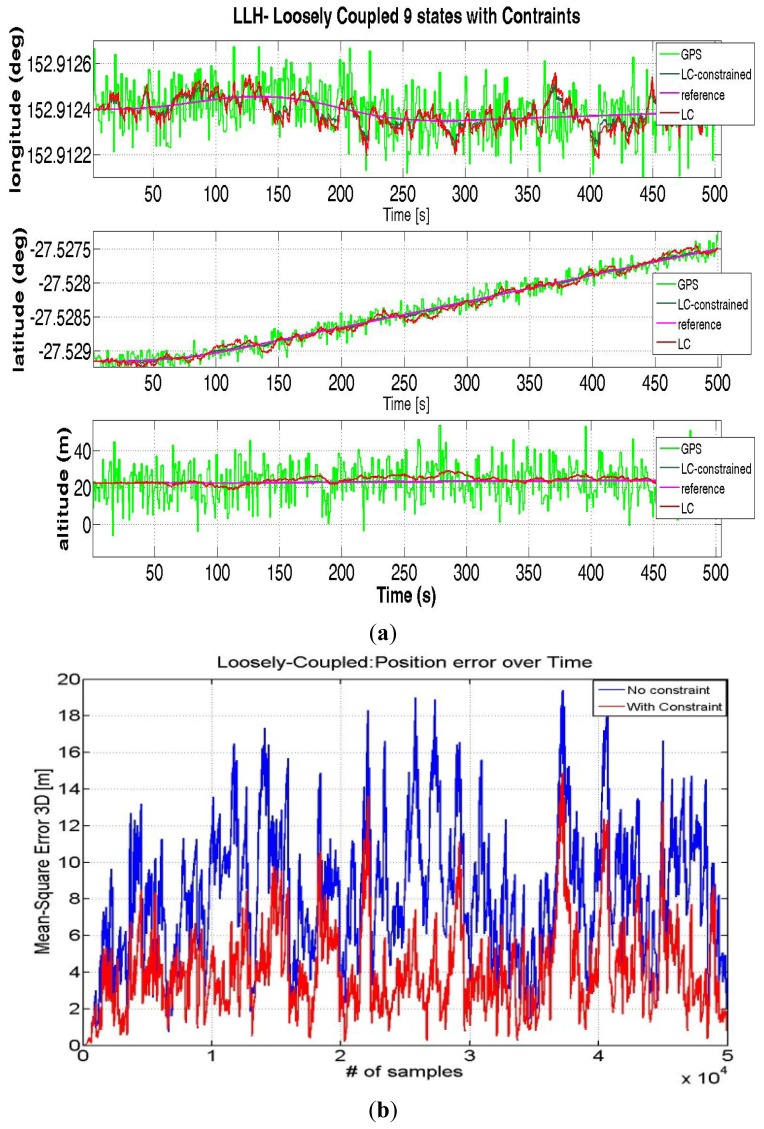
LC with and without constraints (**a**) and Trend of error 3D (**b**).

The position estimates calculated from simulated data are shown in Figure 9, comparing the LC solution using both synthetic and real measurements with and without Map constraints.

It can be seen from Figure 9(b) that the use of additional constraint information can improve the performance. Without constraints the absolute value of the average error is 8.036 m, while with constraints the error is reduced to only 3.056, with an improvement of almost 5 m.

**Figure10.**
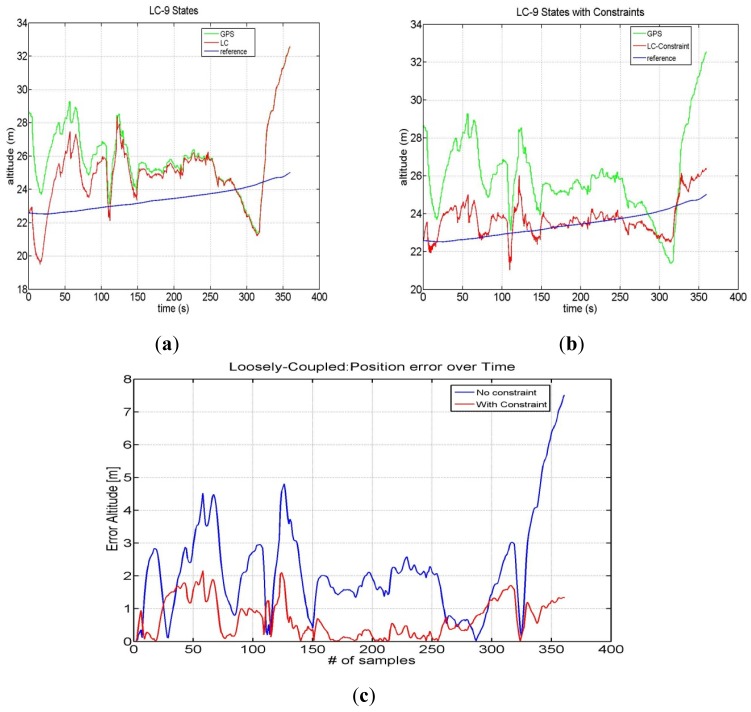
LC without constraints (**a**) and with constraints (**b**). Error trend of altitude (**c**).

The same approach has been used with Google-Maps-sourced altitude information. In this case we have chosen a noise variance of 4 m^2^ for the height constrained filter. The calculated altitude position errors using simulated GPS and INS measurements are shown in Figure 10.

The mean error without additional altitude information is 3.69 m, whereas with constraints the mean error is 1.05 m, thus yielding an improvement of about 2.5 m.

## 4. Loosely Coupled & GPS Outages

**Figure 11.**
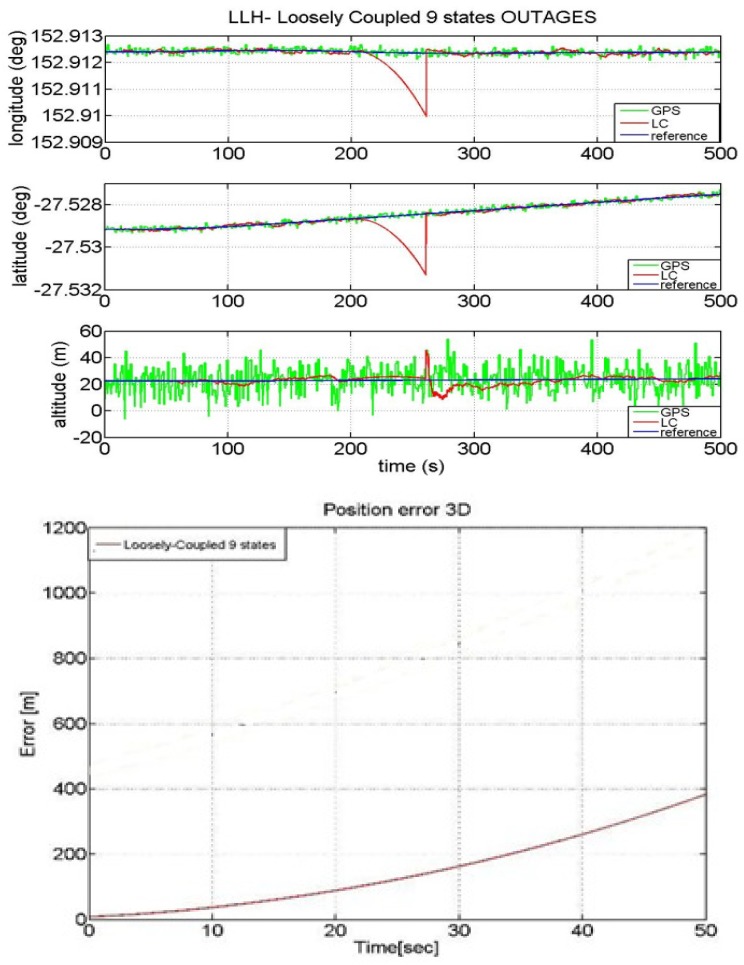
LC in case of GPS outage- Error in case of outage with LC algorithm.

The powerful synergy between the GPS and INS makes the combination of these two navigation technologies a viable position option. GPS, when combined with MEMS inertial devices, can restrict their error growth over time and allows for online estimation of the sensor errors, while the inertial devices can bridge the position estimates when there is no GPS signal reception. Generally speaking, during GPS outages the LC position estimates follow INS-only navigation solution [3]. As a consequence when dealing with low-cost IMU such as the MEMS, the position estimation error grows with time due to the IMU error growth, thus causing a drift in the solution that compromises the long term accuracy of the system. The performance of our LC solution in case of GPS outages of a duration up to 50 seconds is shown in Figure 11.

As shown in Figure 11 the error is about 400 m after a 50 s GPS outage. This quick drifting of the position from the expected one is justified by the low quality of the INS we have used. During the GPS unavailability, the Kalman filter works in prediction mode where the navigation solution leverages on the INS's accelerometers and gyroscopes only. In order to reduce the error further, we employ a time-varying measurement covariance to take into account that the inertial error grows with time.

**Figure 12.**
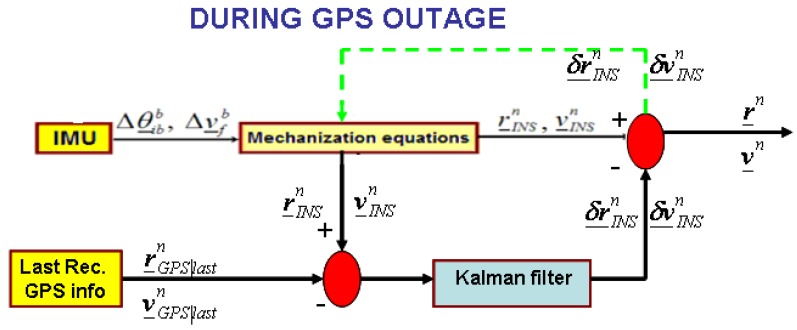
New approach in case of GPS outages.

We have designed a Kalman filter that, by propagating the last GPS information available, corrects the position and velocity estimation as computed by the INS navigation equation only. A block diagram that depicts this strategy is shown in Figure 12.

As we mentioned before, we exploit the last received GPS position information, namely

(26) $$\delta {\underline{\dot{r}}}^{n}=\left[{\underset{═}{F}}_{\text{rr}}\right]+\underline{w}$$

The covariance matrix *Q_K_* associated with the discrete-time noise vector *w̲* is the same as in (16). Concerning on the noise covariance matrix of the states *Q͇*, it can be written as:

(27) $$\underset{═}{Q}=\text{diag}({\underline{q}}_{a})$$

The measurement vector *z̲* becomes:

(28) $$\underline{z}=\left[\delta {\underline{R}}^{n}\right]=\left[{\underline{r}}_{\text{INS}}^{n}-{\underline{r}}_{\text{GPS}|\text{Last}}^{n}\right]=\left[\begin{array}{c} \varphi_{\text{INS}}-\varphi_{\text{GPS}|\text{Last}}\\ \lambda_{\text{INS}}-\lambda_{\text{GPS}|\text{Last}}\\ h_{\text{INS}}-h_{\text{GPS}|\text{Last}} \end{array}\right]$$

The output mapping matrix is given by:

(29) $$\underset{═}{H}=\left[\begin{array}{ccc} R_{M}+h & 0 & 0\\ 0 & (R_{N}+h)\cos\varphi & 0\\ 0 & 0 & 1 \end{array}\right]$$

The noise covariance corresponding to (28) is:

(30) $$\underset{═}{R}=\left[\begin{array}{ccc} {\left(R_{M}+h\right)}^{2}\cdot \sigma_{\varphi }^{2}\cdot K(t) & 0 & 0\\ 0 & {\left(R_{N}+h\right)}^{2}\cdot \cos^{2}\varphi \cdot \sigma_{\lambda }^{2}\cdot K(t) & 0\\ 0 & 0 & \sigma_{h}^{2}\cdot K(t) \end{array}\right]$$

**Figure 13.**
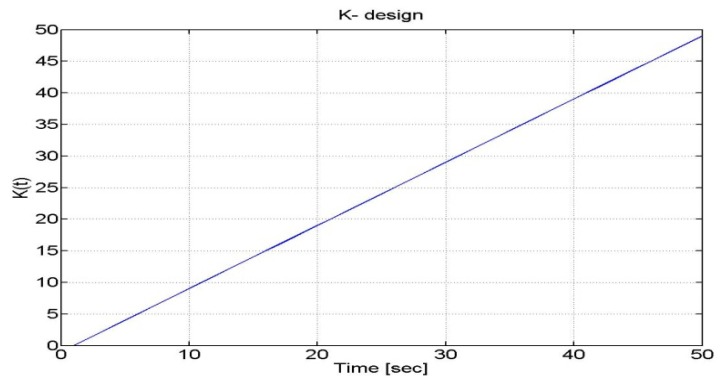
K(t) linear profile.

The time-varying measurement noise covariance results in a time-varying gain. At the beginning of a GPS outage, the GPS estimates are weighted more than the INS outputs. As long as the outage time increases, such weight is decreased. An example of with a linear trend over time is depicted in Figure 13.

**Figure 14.**
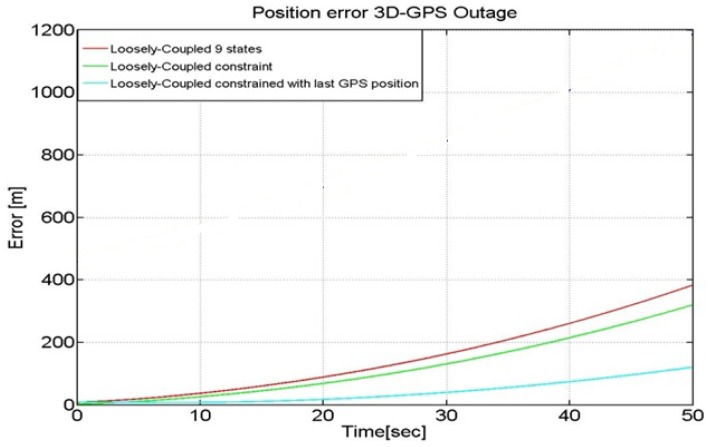
LC in case of GPS outage with weigthening of the last available GPS solution.

The performance resulting from the use of a time-varying measurement noise covariance yields is shown in Figure 14.

It can be seen from Figure 14 that the use of a time-varying measurement covariance can provide a reduction of the error that now has a magnitude of about 100 m after a 50 s GPS outage with respect to the cases of traditional GPS/INS loosely coupled approaches (*i.e.*, with and without constraints). In this analysis the constrained LC has been designed to add boundaries on the altitude axis only.

For the real scenario we have developed a similar Kalman filter that works by using the last available velocity information of GPS. The state-space matrices for the Kalman filter are:

(31) $$\delta {\underline{\dot{v}}}^{n}={\underline{F}}_{\text{vv}}+\underline{w}$$

The definition of all the variables in Equations (26)–(31) is the same as in Section 2 for the Equations (10)–(13) respectively.

The G matrix becomes:

(32) $$\underset{═}{G}={\underset{═}{R}}_{b}^{n}$$

and the noise covariance matrix *Q͇* of the model is:

(33) $$\underset{═}{Q}=\text{diag}({\underline{q}}_{a})$$

For the measurement we can write:

(34) $$\underline{z}=\left[\delta {\underline{v}}^{n}\right]=\left[{\underline{v}}_{\text{INS}}^{n}-{\underline{v}}_{\text{GPS}|\text{last}}^{n}\right]$$

The design matrix *H͇* that bounds the measurements to the error-states becomes:

(35) $$\underline{H}=\left[\begin{array}{ccc} 1 & 0 & 0\\ 0 & 1 & 0\\ 0 & 0 & 1 \end{array}\right]$$

and thus the noise covariance is:

(36) $$\underset{═}{R}=\left[\begin{array}{ccc} \sigma_{\text{VN}}^{2}\cdot \alpha (t) & 0 & 0\\ 0 & \sigma_{\text{VE}}^{2}\cdot \alpha (t) & 0\\ 0 & 0 & \sigma_{\text{VD}}^{2}\cdot \alpha (t) \end{array}\right]$$

α(t) defines the rate at which the noise covariance matrix (referred to the measurements) increases over time.

**Figure 15.**
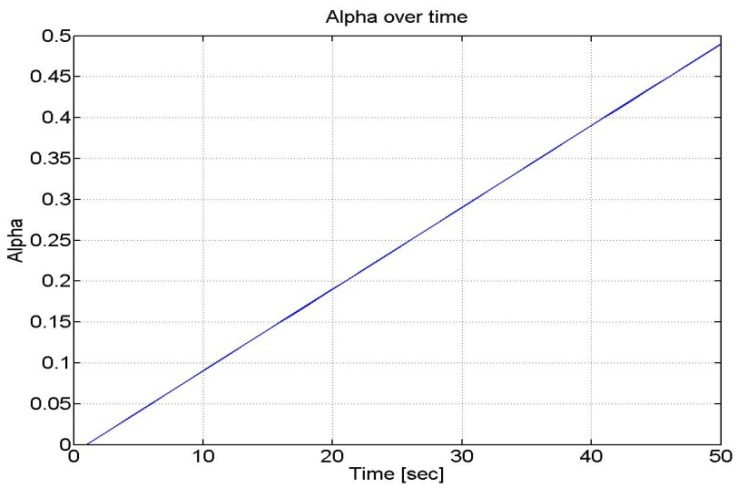
α design for Kalman filter.

We assumed that the parameter α increases very slowly with time (as the velocity of the user was almost constant) as shown in Figure 15.

**Figure 16.**
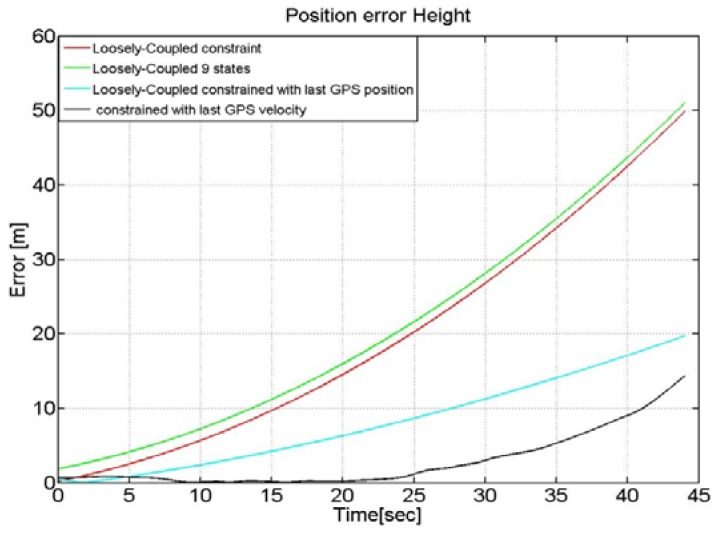
LC in case of GPS outage with weighting strategies.

We have run again the LC solution after having included the modified noise covariance matrix in the Kalman filter design. In particular, Figure 16 highlights the altitude error by using different LC integration approaches. It follows from Figure 16 that the two LC solutions that adopt an adaptive measurements' noise covariance matrix can provide significant improvement during GPS outages.

For example the first method that uses the last user's position information, as provided by the GPS module, gives an error of about 20 m after 45 s of outage with a reduction of almost 30 m with respect a traditional un-constrained LC algorithm that is able to exploit the GPS data only when available and relying on the INS-only navigation during the period of GPS unavailability. The Kalman filter, that exploits the last received update of the GPS velocity and the time-varying weights, provides the best performance with an error after 40 s of only 12 m.

## 5. Conclusions

This paper addresses the subject of loosely-coupled GPS/INS integration. In particular, we have designed a nine states Kalman filter that gives a correction to inertial derived position, velocity and Euler angles by exploiting the available GPS measurements. We have demonstrated the performance of this approach using simulated and real measurements.

In order to improve the LC performance, a constrained approach has been described. Use of maps or altitude constraints can provide benefits in the accuracy of the navigation solution. For example, with simulated measurements, the three-dimensional root-mean-square error (latitude, longitude and altitude) has been reduced of 5m. In the case of real measurements, a 2.5 m reduction in altitude error has been observed.

In addition, we have examined the performance of loosely-coupled integration solutions when GPS outages occur. When GPS information is not available we rely on INS estimates. In order to improve the error during outage times we have developed two solutions to improve performance that exploits an adaptive Kalman filter whose measurement's noise covariance varies over time according to the last GPS update. From the results, it is observed that using the last received GPS position and velocity information can lead to decrease the position error between 30 m and 40 m when a 50-s-long GPS outage occurs.

## Figures and Tables

**Table 1.** Typical characteristics of a low-cost MEMS IMU.

**Gyroscope (Angular Rate)**	Noise (ARW) $\left[{}^{\circ}/\sqrt{h}\right]$
	3

**Accelerometer**	Noise (VRW) $\left[\omega g/\sqrt{Hz}\right]$
	1000

**Table 2.** Simulated measurement variances.

**GPS** Noise Variance (R)	Position [m^2^]
	100

	Velocity [m/sec]^2^
	10

**Table 3.** Noise covariance features.

**GPS** Noise Variance (R)	Position [m^2^]
	10

	Velocity [m/s]^2^
	0.2
